# Risk analysis of cardiovascular mortality after gastric cancer diagnosis: a large population-based study

**DOI:** 10.3389/fcvm.2025.1459151

**Published:** 2025-04-22

**Authors:** Qiang Zhao, Qiaohong Zhou, Jiayue Dong, Qiang Tong

**Affiliations:** ^1^ Department of Cardiology, Affiliated Jinhua Hospital, Zhejiang University School of Medicine, Jinhua, Zhejiang, China; ^2^ Department of Pharmacy, Affiliated Jinhua Hospital, Zhejiang University School of Medicine, Jinhua, Zhejiang, China; ^3^ Department of Gastroenterology, Affiliated Jinhua Hospital, Zhejiang University School of Medicine, Jinhua, Zhejiang, China

**Keywords:** cardiovascular mortality, gastric cancer, risk predictor, competing risk models, cardio-oncology teams

## Abstract

**Background:**

The increasing prevalence of cardiovascular mortality is becoming a significant worry for individuals who have survived cancer. The aim of this study is to investigate the dynamic trend of cardiovascular death in patients with gastric cancer (GC) and identify the risk factors associated with cardiovascular disease (CVD)-specific mortality in non-metastatic GC patients.

**Methods:**

In the present study, 29,324 eligible patients diagnosed with primary GC were collected from the Surveillance, Epidemiology, and End Results (SEER) database. Standardized mortality ratios (SMRs) adjusted by age, gender, calendar year, and race were calculated. Fine-Gray's competing risk models were taken to identify the prognostic factors of cardiovascular death in GC patients.

**Results:**

There were 1083 (5.2%) cardiovascular deaths among 20,857 patients with local/regional GC, and 76 (0.9%) cardiovascular deaths among 8,467 patients with metastatic GC. The SMRs of CVD-specific mortality continuously increased since the 1975s throughout the 2015s. The competing risk models showed that age (>75 years vs. 0–50 years, HR: 6.602, 95% CI: 4.356–10.006), T stage (T4 vs. T1, HR:0.524, 95% CI: 0.370–0.741), N stage (N3 vs. N0, HR: 0.557, 95% CI: 0.343–0.903), surgery (Yes vs. No, HR: 0.551, 95% CI: 0.461–0.659), and radiotherapy (Yes vs. No, HR: 1.011, 95% CI: 1.011–1.437) were predictive of CVD-specific mortality. Furthermore, based on the results of the competing risk analyses, a nomogram was constructed to predict the probability of CVD-specific mortality for local/regional GC patients.

**Conclusion:**

Our study demonstrated the dynamic trend of cardiovascular death in GC patients, and identified prognostic risk predictors, highlighting the importance cardio-oncology teams in offering comprehensive care and long-term follow-up for GC patients.

## 1 Introduction

Gastric cancer (GC) ranks as the fifth most prevalent malignancy and the third deadliest cancer worldwide, with over 1,000,000 new cases and 783,000 deaths annually, accounting for approximately 1 in every 12 mortalities globally (1, 2). Owing to the advancements in early detection and innovative treatments, the life expectancy of GC patients has significantly improved since the 1990s and early 2000s (3–5). With the growing population of GC survivors, understanding the precise causes of mortality among these individuals is vital for prioritizing healthcare interventions during survivorship.

Previous research on the causes of mortality among cancer patients has revealed that cancer survivors face an elevated risk of cardiovascular diseases (CVDs), stemming either from the toxicities of cancer treatments or from shared lifestyle (6–8). Several studies have highlighted the close correlation between increased cardiovascular mortality risk and cancer treatments in GC patients (9–11). Therefore, ensuring proper cardiology care for GC survivors is becoming increasingly crucial. However, the prognostic risk factors for predicting CVD-specific death in GC patients remain unknown, and effective clinical guidelines are still lacking (9).

Underestimating the increased risk of CVD faced by GC survivors may lead to missed opportunities for early intervention. To gain a deeper understanding of cardiovascular mortality among GC survivors, we conducted a comprehensive investigation based on data from the Surveillance, Epidemiology, and End Results (SEER) database. Our goals were to elucidate historical trends in cardiovascular mortality and identify prognostic risk factors for cardiovascular mortality in GC patients.

## 2 Methods

### 2.1 Data source

The SEER database encompasses approximately 28% of the general US population and collects demographic, clinical information, and survival data (12). Permission to access the database was obtained by signing and submitting a SEER Research Data Agreement form via email. The SEER∗Stat software was used to access the 18 Registry Research Datasets (2000–2015, with additional treatment fields; November 2018 sub). As all data extracted from the SEER database were de-identified and anonymized before release, local ethics approval was not required for this study.

### 2.2 Study population and definition

All cases diagnosed with GC as their first primary malignancy between January 1, 2000, and December 31, 2015, were included. Patients diagnosed solely through death certificates or autopsy, with missing information on SEER Summary Stage 2000 (local/regional or distant), unknown information on pathological grade, surgery status, adjuvant treatment status, or cause of death were excluded. Follow-up time was defined as the period from diagnosis to the death date, the end of the study period (December 31, 2020), or the date of last contact, whichever occurred first. Cardiovascular mortality encompassed mortality caused by aortic aneurysm/dissection, atherosclerosis, heart diseases (including acute myocardial infarction or other ischemic heart diseases), cerebrovascular diseases, other diseases of capillaries, arteries, and arterioles, as well as hypertension without heart disease (13, 14). Demographic and clinical information of interest, including age at diagnosis, year of diagnosis, gender (male and female), ethnicity, SEER histologic stage (local/regional and distant), grade, histology type, treatment (surgery, chemotherapy, and radiotherapy), and survival months, were collected for analysis.

### 2.3 Statistical analysis

Statistical analyses were performed using SPSS (version 25.0) or R (version 3.6.1). Descriptive statistics were used to describe the distribution of baseline characteristics. Categorical variables were presented as percentages and compared using Fisher's exact test, while continuous variables were summarized as median values and evaluated using the Kruskal–Wallis rank sum test. Standardized mortality ratios (SMRs), adjusted by age, gender, calendar year, and race, were calculated to compare cardiovascular death rates in our study population with those of the general population. SMRs were defined as the ratios of observed-to-expected deaths due to CVD, with 95% confidence intervals (95% CIs) calculated using exact methods (15, 16). Univariate and multivariate Fine-Gray's competing risk models were employed to calculate CVD-specific hazard ratios (HRs) to evaluate the relative association between prognostic factors and the risk of cardiovascular death (with non-cardiovascular mortality as a competing risk) (13). All tests were two-tailed, with a *p*-value of <0.05 considered statistically significant.

## 3 Results

### 3.1 Baseline characteristics

A total of 29,324 patients diagnosed with primary GC were included in our study, with 10,688 female patients and 18,636 male patients. Of the included patients, the majority were diagnosed with GC at age >50 (*n* = 25,699, 87.6%) and white (*n* = 20,213, 68.9%). Most patients (*n* = 20,857, 71.1%) were diagnosed with local/regional GC, while only 28.9% (*n* = 8,467) were diagnosed with distant GC. The majority of patients received surgical treatment, due to which a reduction in death was achieved compared to patients receiving non-surgical treatment (death rate: 60.8% vs. 90.9%). Given that most patients with distant GC had different oncological characteristics and received different treatment strategies compared to those with local/regional GC, we analyze the cohort of distant GC separately. Table 1 summarized the baseline clinical characteristics of the included patients by tumor stage.

**Table 1:** The baseline clinical characteristics of enrolled GC patients at diagnosis.

Characteristics	Overall (*n* = 29,324)	Local/regional GC (*n* = 20,857)	Distant GC (*n* = 8,467)	*P*-value
Survival time, month (IQR)	17 (7, 43)	24 (10, 56)	8 (3,17)	
Age				
<50	3,625 (12.4%)	2,142 (10.3%)	1,483 (17.5%)	<0.0001
[50, 65)	9,118 (31.1%)	6,102 (29.3%)	3,016 (35.6%)	
[65, 75)	7,912 (27.0%)	5,787 (27.7%)	2,125 (25.1%)	
≥75	8,669 (29.6%)	6,826 (32.7%)	1,843 (21.8%)	
Race				
White	20,213 (68.9%)	14,160 (67.9%)	6,053 (71.5%)	<0.0001
Black	3,896 (13.3%)	2,752 (13.2%)	1,134 (13.4%)	
Other	5,215 (17.8)	3,935 (18.9%)	1,280 (15.1%)	
Sex				
Female	10,688 (36.4%)	7,711 (37.0%)	2,977 (35.2%)	0.004
Male	18,636 (63.6%)	13,146 (63.0%)	5,490 (64.8%)	
Site				
Cardia	9,517 (32.5%)	6,565 (31.5%)	2,952 (34.9%)	<0.0001
Gastric antrum	6,325 (21.6%)	4,880 (23.4%)	1,445 (17.1%)	
Body of stomach	2,700 (9.2%)	1,912 (9.2%)	788 (9.3%)	
Lesser curvature of stomach	2,635 (9.0%)	2,047 (9.8%)	598 (7.1%)	
Other	8,137 (27.7)	5,453 (26.1%)	2,684 (31.7%)	
Grade				
I	1,620 (5.5%)	1,420 (6.8%)	200 (2.4%)	<0.0001
II	7,916 (27.0%)	6,047 (29.0%)	1,869 (22.1%)	
III	19,097 (65.1%)	12,913 (61.9%)	6,184 (73.0%)	
IV	691 (2.4%)	477 (2.3%)	214 (2.5%)	
T stage				
T1	8,387 (28.6%)	6,412 (30.7%)	1,975 (23.3%)	<0.0001
T2	11,674 (39.8%)	9,101 (43.6%)	2,573 (30.4%)	
T3	3,560 (12.1%)	3,898 (18.7%)	1,532 (18.1%)	
T4	1,446 (4.9%)	1,446 (6.9%)	2,387 (28.2%)	
N stage				
N0	13,066 (44.6%)	10,410 (49.9%)	2,656 (31.4%)	<0.0001
N1	11,252 (38.4%)	7,213 (34.6%)	4,039 (47.7%)	
N2	3,560 (12.1%)	2,394 (11.5%)	1,166 (13.8%)	
N3	1,446 (4.9%)	840 (4.0%)	606 (7.2%)	
Treatment received				
No	3,089 (10.5%)	1,672 (8.0%)	1,417 (16.7%)	<0.0001
Yes	26,235 (89.5%)	19,185 (92.0%)	7,050 (83.3%)	
Surgery				
No	9,636 (32.9%)	4,154 (19.9%)	5,482 (64.7%)	<0.0001
Yes	19,688 (67.1%)	16,703 (80.1%)	2,985 (35.3%)	
Radiotherapy				
No	19,682 (67.1%)	13,429 (64.4%)	6,253 (73.9%)	<0.0001
Yes	9,642 (32.9%)	7,428 (35.6%)	2,214 (26.1%)	
Chemotherapy				
No	13,139 (44.8%)	10,431 (50.0%)	2,708 (32.0%)	<0.0001
Yes	16,185 (55.2%)	10,426 (50.0%)	5,759 (68.0%)	

### 3.2 Cause-specific mortality among patients with GC

**Figure 1:**
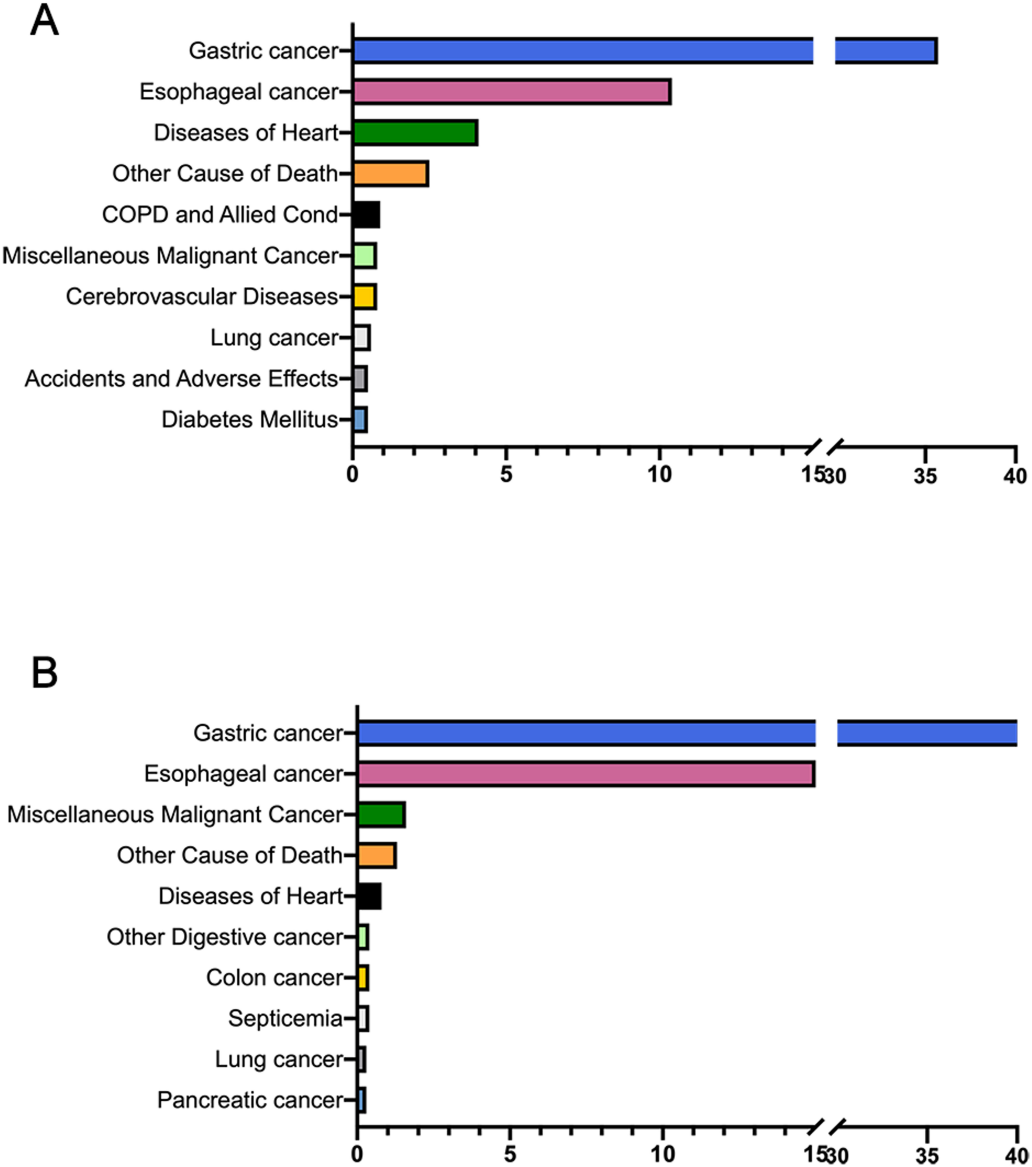
The top ten causes of death among patients with local/regional GC **(A)** and distant GC **(B)**.

The main causes of mortality among patients with local/regional GC and patients with distant GC were summarized (Figure 1). Specifically, for patients with local/regional GC, GC was still the leading cause of mortality (*n* = 7,438, 35.7%), followed by esophageal cancer (*n* = 2,170, 10.4%) and heart diseases (*n* = 846, 4.1%). It is noteworthy that plurality (77.4%) of cardiovascular deaths in patients with GC were caused by heart diseases. For patients with distant GC, mortality from GC (*n* = 5,529, 65.3%) composed the majority of deaths, while non-cancer causes and other cancers were less common.

As evidenced in Table 1, there is a striking disparity in surgical intervention rates between distant and local/regional GC patients, with only 35.3% of distant GC patients undergoing surgery compared to 80.1% of local/regional cases. Furthermore, the proportion of distant GC patients not receiving any medical treatment (16.7%) is notably higher than that of local/regional patients (8.0%). Recognizing the substantial disparities in treatment approaches and survival outcomes between patients with distant metastatic GC and those with local/regional advanced disease (Table 1 and Figure 1), we excluded the distant metastatic cohort from subsequent SMR analyses to ensure more accurate and clinically relevant comparisons. We used SMRs to compared CVD-specific death in our study population with that of the general population (Table 2). Overall, the SMR between patients with local/regional GC was 2.10 (95% CI: 2.03–2.17), with 2.86 (95% CI: 2.70–3.03) for female patients, 3.52 (95% CI: 3.36–3.70) for male patients; 4.38 (95% CI: 3.98–4.81) for black ethnicity, 2.94 (95% CI: 2.81–3.07) for white ethnicity, 3.80 (95% CI: 3.44–4.19) for other (Asian/Pacific Islander, American Indian/Alaska Native). The SMRs of older adult patients were gradually decreased compared to those of young patients (<50 years, SMR: 53.64, 95% CI: 34.71–79.19; >75 years, SMR: 2.54, 95% CI: 2.43–2.65).

**Table 2:** Standardized mortality ratios among local/regional GC patients by demographic and clinic characteristics.

Variables	SMR (95% CI)	95%CI		Excess risk	CVD-specific mortality rate (per 100,000 person-years)
		Lower	Up		
Age					
<50	53.64	34.71	79.19	1,684.53	145.64
[50,65)	16.01	13.99	18.24	1,608.60	1,317.17
[65,75)	9.26	8.50	10.08	1,853.96	2,593.47
≥75	2.54	2.43	2.65	1,366.28	9,289.95
Race					
White	2.94	2.81	3.07	1,422.27	9,421.67
Black	4.38	3.98	4.81	1,956.41	1,727.93
Other	3.80	3.44	4.19	1,370.07	2,145.89
Sex					
Female	2.86	2.70	3.03	1,318.93	6,014.46
Male	3.52	3.36	3.70	1,627.48	7,331.78
Site					
Cardia	4.52	4.18	4.87	1,977.62	2,649.83
Gastric antrum	2.68	2.49	2.89	1,281.41	3,447.19
Body of stomach	2.87	2.57	3.20	1,225.95	1,753.36
Lesser curvature of stomach	2.74	2.43	3.08	1,263.18	1,437.82
Other	3.33	3.12	3.56	1,538.09	4,058.04
Grade					
I	2.90	2.56	3.27	1,262.71	1,343.47
II	2.95	2.75	3.16	1,419.11	3,775.15
III	3.58	3.38	3.80	1,689.14	4,873.21
IV	2.92	2.17	3.85	1,361.21	241.52
T stage					
T1	3.36	3.13	3.60	1,694.12	3,294.74
T2	4.21	3.88	4.57	2,128.72	2,135.40
T3	5.18	4.42	6.03	2,474.46	534.79
T4	7.14	5.19	9.58	3,590.81	105.37
N stage					
N0	3.26	3.07	3.46	1,638.43	4,459.30
N1	5.60	5.06	6.18	2,632.67	1,235.39
N2	7.76	6.23	9.55	3,253.13	238.32
N3	11.59	7.18	17.72	6,072.64	31.60
Surgery					
No	5.85	5.41	6.33	3,404.01	1,563.87
Yes	2.84	2.72	2.96	1,232.68	11,758.85
Radiotherapy					
No	3.12	3.00	3.24	1,503.90	11,349.83
Yes	3.99	3.60	4.42	1,400.48	1,996.41
Chemotherapy					
No	2.93	2.81	3.06	1,393.26	10,528.49
Yes	4.73	4.37	5.10	1,844.06	2,817.74

**Figure 2:**
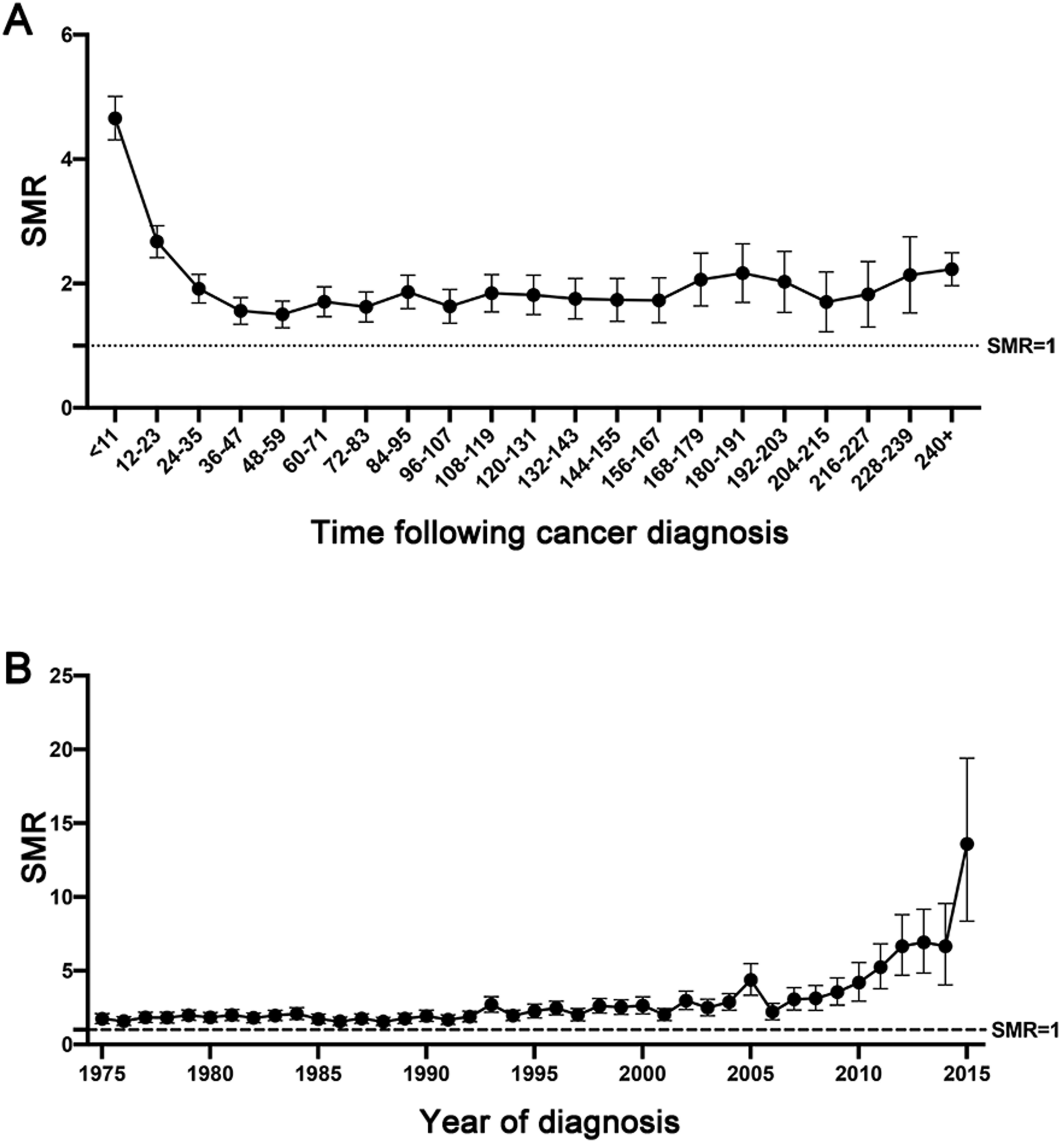
The dynamic trend of SMRs of cardiovascular death among local/regional GC at different latency periods **(A)**, and at different year of diagnosis **(B)**.

Notably, the CVD-specific death risk among local/regional GC patients was high within the first year following GC diagnosis (<12 months, SMR: 4.65, 95% CI: 4.31–5.01), and it remained elevated compared to that of the general population throughout the entail follow-up period (12–60 months, SMR: 1.94, 95% CI: 1.83–2.06; >120 months, SMR: 1.93, 95% CI: 1.82–2.04, Figure 2A). In addition, we observed that the risk of CVD-specific mortality continuously increased since the 1975s throughout the 2015s (the 1975s, SMR: 1.73, 95% CI: 1.41–2.09; the 2000s, SMR: 2.61, 95% CI: 2.08–3.23; the 2015s, SMR: 13.04, 95% CI: 8.36–19.41, Figure 2B).

### 3.3 Risk factors for CVD-associated mortality

**Figure 3:**
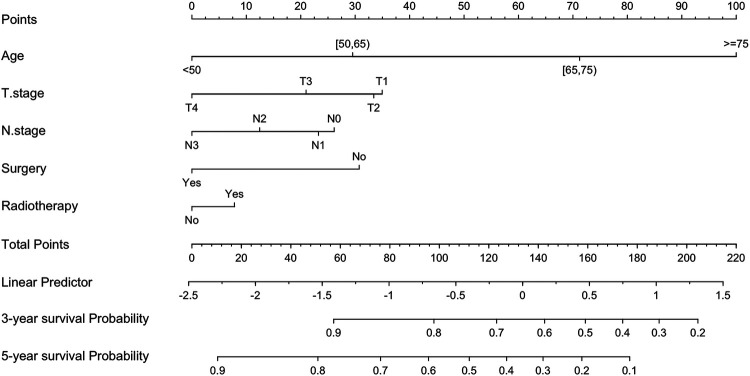
Nomogram for predicting CVD-specific survival in patients with local/regional GC.

Fine-Gray's competing risk analyses were applied to identify the risk predictors for cardiovascular deaths among local/regional GC patients (Table 3). The results of the univariate competing risk model indicated that age, differentiation grade, T stage, N stage, surgery of the primary tumor, radiotherapy, and chemotherapy were significantly related to the prognosis of CVD-specific mortality. Subsequently, these factors were assessed using a multivariate competing risk model, and found that except for differentiation grade and chemotherapy, age at diagnosis (>75 years old vs. 0–50 years old, HR: 6.602, 95% CI: 4.356–10.006, *p* < 0.001), T stage (T4 vs. T1, HR:0.524, 95% CI: 0.370–0.741, *p* < 0.0001), N stage (N3 vs. N0, HR: 0.557, 95% CI: 0.343–0.903, *p* = 0.018), surgery (Yes vs. No, HR: 0.551, 95% CI: 0.461–0.659, *p* < 0.0001), and radiotherapy (Yes vs. No, HR: 1.011, 95% CI: 1.011–1.437, *p* = 0.037) were predictive of CVD-specific mortality. Detailed information regarding the predictors of CVD-specific death in the study cohort is presented in Table 3. Further, based on the results of Fine-Gray's competing risk analyses, a nomogram was constructed to predict the probability of CVD-specific mortality for local/regional GC patients (Figure 3).

**Table 3:** Fine-Gray's competing risk model analysis for CVD-specific death in patients with local/regional gastric cancer.

Variables	Univariate				Multivariate			
	Hazard ratio	95% CI		*P*	Hazard ratio	95% CI		*P*
		Lower	Up			Lower	Up	
Age								
<50	Ref.							
[50, 65)	1.870	1.210	2.900	<0.0001	1.729	1.117	2.677	0.014
[65, 75)	4.330	2.850	6.570	<0.0001	3.644	2.395	5.544	<0.0001
≥75	8.900	5.930	13.370	<0.0001	6.602	4.356	10.006	<0.0001
Race								
White								
Black	1.176	0.995	1.390	0.057				
Other	0.855	0.726	1.010	0.062				
Sex								
Female								
Male	0.896	0.793	1.010	0.075				
Site								
Other								
Cardia	0.805	0.684	0.948	0.009				
Gastric antrum	1.210	1.032	1.418	0.019				
Body of stomach	0.977	0.778	1.227	0.840				
Lesser curvature of stomach	0.877	0.796	1.103	0.260				
Grade								
I								
II	0.895	0.720	1.113	0.320	0.982	0.789	1.223	0.870
III	0.590	0.478	0.729	<0.0001	0.844	0.678	1.050	0.130
IV	0.634	0.401	1.002	0.052	0.887	0.560	1.404	0.610
T stage								
T1								
T2	0.697	0.613	0.794	<0.0001	0.981	0.848	1.136	0.080
T3	0.462	0.380	0.561	<0.0001	0.777	0.621	0.972	0.027
T4	0.338	0.241	0.475	<0.0001	0.524	0.370	0.741	<0.0001
N stage								
N0								
N1	0.622	0.543	0.712	<0.0001	0.898	0.768	1.050	0.180
N2	0.444	0.348	0.567	<0.0001	0.708	0.544	0.922	0.010
N3	0.321	0.201	0.513	<0.0001	0.557	0.343	0.903	0.018
Surgery								
No								
Yes	0.374	0.327	0.427	<0.0001	0.551	0.461	0.659	<0.0001
Radiotherapy								
No								
Yes	0.542	0.471	0.623	<0.0001	1.205	1.011	1.437	0.037
Chemotherapy								
No								
Yes	0.800	0.695	0.992	0.002	1.025	0.877	1.198	0.750

## 4 Discussion

To our knowledge, this population-base cohort study represents the most comprehensive and largest characterization of cardiovascular death among patients with GC. Our findings corroborate that cardiovascular mortality remains a challenge for individuals diagnosed with GC. Additionally, the Fine-Gray's competing risk analyses were utilized to identify several predictors for CVD-specific mortality, including age, gender, T stage, N stage, primary site, differentiation grade, surgery, radiotherapy, and chemotherapy. Our findings align with previous reports on cardiovascular risks in other malignancies (7, 17–19), highlighting the critical need for sustained cardiovascular care throughout the survivorship period.

With the widespread implementation of GC screening and advancements in cancer treatment, the survival rates of GC are improving, which indicates the importance of the management of comorbidities for survivors (19, 20). Recently, Lou et al. conducted a retrospective analysis of the causes of death among 42,813 GC patients. The results revealed that GC (66.7%) was the primary cause of death among these patients, followed by other types of cancer (17.6%). Additionally, among non-cancer causes of death, heart disease (5.7%) ranked first, with cerebrovascular disease (1.4%) closely following (21). Similarly, in the present study, GC was still the main cause of death for patients with GC, especially for those with metastatic status. Notably, among patients with local/regional GC, CVD ranked third among all the causes of death, with its proportion increasing over the follow-up period.

Evaluating SMRs offers important population-level data to screen GC patients who are at risk for elevated CVD-specific death (7, 17). In line with previous studies, a higher SMR was observed among patients with younger age of diagnosis, both historically and in the era of modern treatment (7). The risk for CVD-specific death occurred was greatest within the first year of GC diagnosis and decreased year by year during follow-up, which had been reported in other cancer studies (7, 22) and this may be likely due to aggressive cancer treatment shortly after GC diagnosis (23). This trend may also stem from the fact that patients with the most severe co-existing cardiovascular diseases are at higher risk of cancer treatment and are more likely to die early after cancer discovery (24, 25). Furthermore, compared to patients diagnosed in previous years, those recently diagnosed with GC faced a higher risk of CVD-specific death. This could be attributed to the shorter follow-up time for recently diagnosed patients. Since SMRs of cardiovascular death tended to be highest within the first few years of cancer discovery, SMRs for recently diagnosed patients were partially skewed and higher than those for patients diagnosed in prior years. Additionally, studies revealed that the emerging novel treatments (e.g., targeted therapy, immune checkpoint inhibitors) could cause severe cardiac toxicity (26, 27), which also contributed to the explanation of the elevated SMRs of cardiovascular mortality in recent years.

In the present study, Fine-Gray's competing risk analyses were conducted to identify risk factors for CVD-specific mortality among patients with local/regional GC. Specifically, age at diagnosis emerged as the predominant risk predictor for cardiovascular death, and the older adult patients faced higher risks of cardiovascular death (28). Regarding the histological features of tumor, patients with advanced tumor stage (i.e., T3/T4 status or N3 status) exhibited a lower risk of cardiovascular mortality. A possible explanation of this finding was that those with advance tumor stage were more likely to die of cancer shortly after GC diagnosis.

In terms of cancer treatment, patients who underwent surgery (such as subtotal or total gastrectomy) had lower risks of cardiovascular death in our study. The possible mechanisms explaining the decreased risks of CVD mortality after surgery were various, including reductions in body weight, subcutaneous fat area, and visceral fat area, and the improvement of glycemic control and metabolic profile (29, 30). Chemotherapy and radiotherapy were corroborated to be effective for GC treatment in various respects, such as delaying the metastasis of GC, decreasing the risk of local recurrence, and so on (31, 32). Nevertheless, in this study, we observed that radiotherapy was associated with the elevated risks of CVD-specific mortality among GC patients, indicating the necessity of detailed cardiovascular evaluation before radiotherapy. Moreover, the accelerated development of innovative therapeutic approaches, such as immunotherapy and targeted therapies, has significantly enhanced clinical outcomes for numerous gastric cancer (GC) patients. Further research is anticipated to delve deeply into how these contemporary treatments influence cardiovascular mortality rates.

Our study has several inherent limitations that merit careful consideration. The retrospective nature of the SEER database analysis inherently restricts its capacity to establish definitive causal relationships. A particularly notable limitation pertains to the evaluation of radiotherapy's association with increased cardiovascular mortality, as the absence of detailed dosimetric parameters and specific radiation technique information significantly impedes the formulation of clinically actionable conclusions. Other limitations align with previous comprehensive evaluations of the SEER database's inherent biases and constraints (33). Specifically, potential misclassification of cardiovascular disease-related mortality in death certificate data may lead to inaccuracies in cardiovascular disease estimation. Furthermore, the unavailability of comprehensive comorbidity data, including conditions such as diabetes mellitus, precluded our ability to assess their potential impact on cardiovascular mortality risk.

Notwithstanding these limitations, our findings provide valuable insights that underscore the importance of early cardiology involvement in patient care. Future research should focus on two critical areas: (1) establishing optimal protocols for early cardiology assessment in gastric cancer patients, and (2) determining the appropriate intensity of cardiology care in this patient population. These investigations would significantly enhance our understanding of cardiovascular risk management in gastric cancer patients undergoing treatment.

## 5 Conclusion

In summary, this study demonstrated the elevated risk of dying from CVDs in patients with GC and identified age at diagnosis, T stage, N stage, surgery of the primary site, and radiotherapy as potential risk factors for cardiovascular mortality using Fine-Gray's competing risk model. Our study underscores the importance cardio-oncology teams in offering comprehensive care and long-term follow-up for GC patients. However, the optimal integration of cardiovascular care into standard oncology treatment protocols remains an area requiring consensus. In this context, Bonaca et al. have proposed an innovative approach through the establishment of collaborative think tanks to systematically evaluate cardiovascular safety in cancer therapy trials (19). Most importantly, there is an urgent need to establish comprehensive, evidence-based guidelines for standardized cardiovascular care specifically tailored for GC patients. These guidelines should address critical gaps in current practice, including optimal screening protocols, risk stratification methods, and integrated care pathways throughout the cancer treatment continuum. The development of such standards requires collaborative efforts between oncologists and cardiologists to ensure both cancer treatment efficacy and cardiovascular safety.

## Data Availability

The original contributions presented in the study are included in the article/Supplementary Material, further inquiries can be directed to the corresponding author.
